# Complete Dissection of the Interventricular Septum Following Myocardial Infarction

**DOI:** 10.7759/cureus.15443

**Published:** 2021-06-04

**Authors:** Aref Obagi, Satish Tadepalli, Jayant Reddy, Pramil Cheriyath, Arthur Okere

**Affiliations:** 1 Internal Medicine, Jersey Shore University Medical Center, Neptune, USA; 2 Internal Medicine, Hackensack Meridian Health - Ocean Medical Center, Brick, USA; 3 Cardiology, Hackensack Meridian Health - Ocean Medical Center, Brick, USA

**Keywords:** st-elevation myocardial infarction (stemi), ventricular septal defect (vsd), acute cardiogenic pulmonary edema, left ventricular mechanical support, ventricular septal dissection

## Abstract

In this report, we present a case of interventricular septal dissection (IVSD) following inferior wall myocardial infarction (MI) in a 64-year-old patient; the patient ultimately recovered after prompt resuscitation and intervention, despite the high mortality associated with these cases.

A 64-year-old male with a history of hypertension and obesity was brought to the hospital following an episode of syncope at home. He had been experiencing chest tightness over the past few days prior to the admission. On physical exam, he had a heart rate of 72 beats per minute and blood pressure of 73/52 mmHg. His electrocardiogram revealed ST-segment elevations in leads II, III, and aVF. Emergent coronary angiography revealed 100% occlusion of the right coronary artery (RCA) with no collateral supply and 95% stenosis of the left anterior descending (LAD) artery. Aspiration thrombectomy and balloon angioplasty and subsequent stenting of the RCA were performed. Transthoracic echocardiogram with color Doppler was performed, which confirmed the presence of a defect in the septum. Color Doppler demonstrated a clear jet entering the ventricular septum from the left ventricle (LV), with the jet traversing the entire length of the septum through a dissection and entering into the right ventricle (RV), consistent with complete IVSD. The patient subsequently underwent a successful bovine pericardial patch repair of the ventricular septum.

IVSD is a rare anomaly of the IVS. An echocardiogram is a useful tool to establish the diagnosis. The mortality rate after ventricular septal rupture remains high. Fortunately, our patient had interventricular dissection without rupture. Prompt surgical repair remains the choice of treatment for this condition.

## Introduction

Interventricular septal dissection (IVSD) is a rare complication of myocardial infarction (MI), and it is associated with a high mortality rate [1]. The most common cause of IVSD is right sinuses of Valsalva (SOV) aneurism [2]. It is an extremely rare complication of acute MI, and only a few cases of IVSD have been reported in the literature. Other causes of IVSD include infective endocarditis, cardiac surgery, and trauma.

We report a case of IVSD following inferior wall MI in a 64-year-old patient who ultimately recovered after prompt resuscitation and intervention, despite the high mortality rate associated with these cases.

## Case presentation

**Figure 1:**
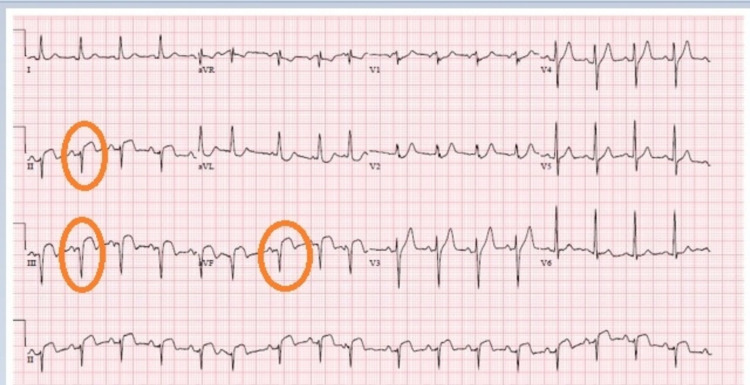
EKG at presentation showing ST-elevations with Q-wave in leads 2-3-aVF (orange ovals) EKG: electrocardiogram

A 64-year-old male with a history of hypertension and obesity was brought to the hospital following an episode of syncope at home. He had experienced chest tightness over the past few days prior to the admission. On physical exam, he was afebrile, with a heart rate of 72 beats per minute and blood pressure of 73/52 mmHg. Cardiac examination revealed a rhythmic S1 and S2, with a III/VI holosystolic murmur at the left lower sternal border. His laboratory examination revealed troponin levels of 17.92 ng/ml. His electrocardiogram revealed ST-segment elevations in leads II, III, and aVF (Figure 1).

**Figure 2:**
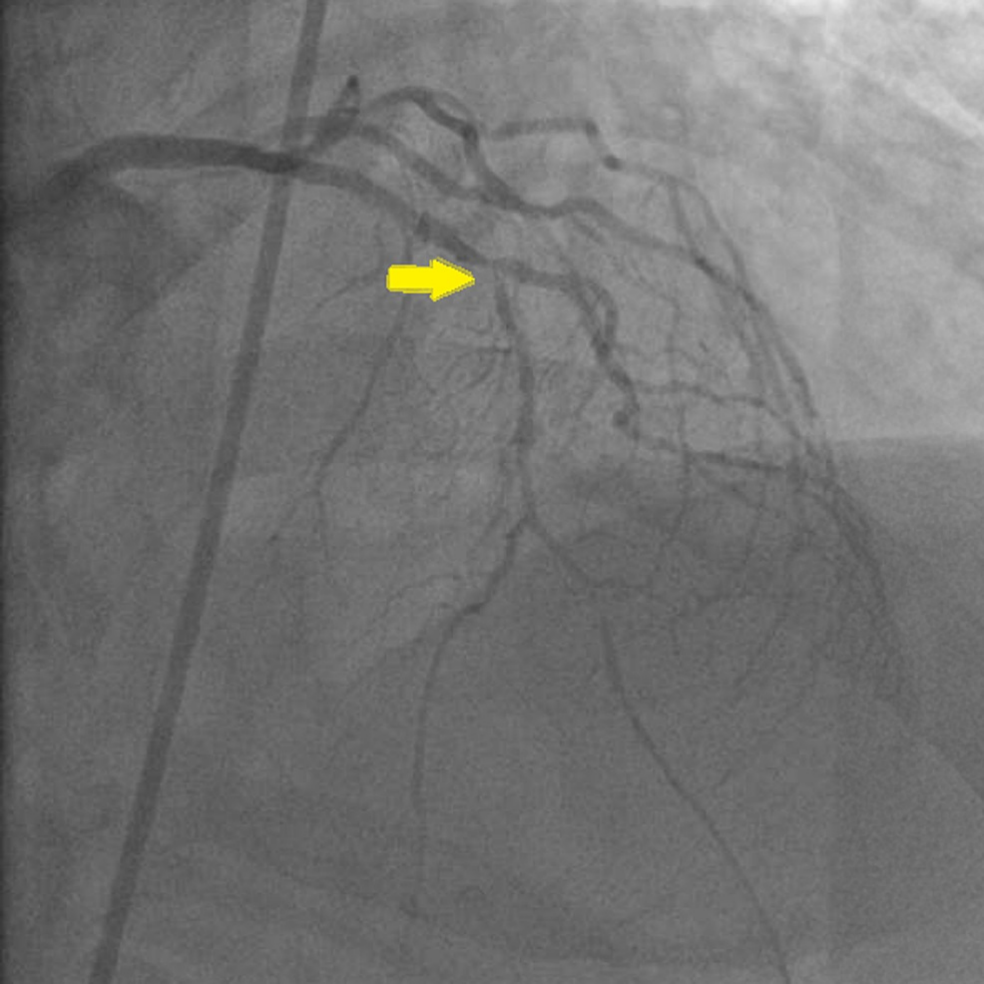
AP/CRA view of the LAD showing 95% stenosis in mid-segment (arrow) AP: anterior-posterior; CRA: cranial; LAD: left anterior descending

**Figure 3:**
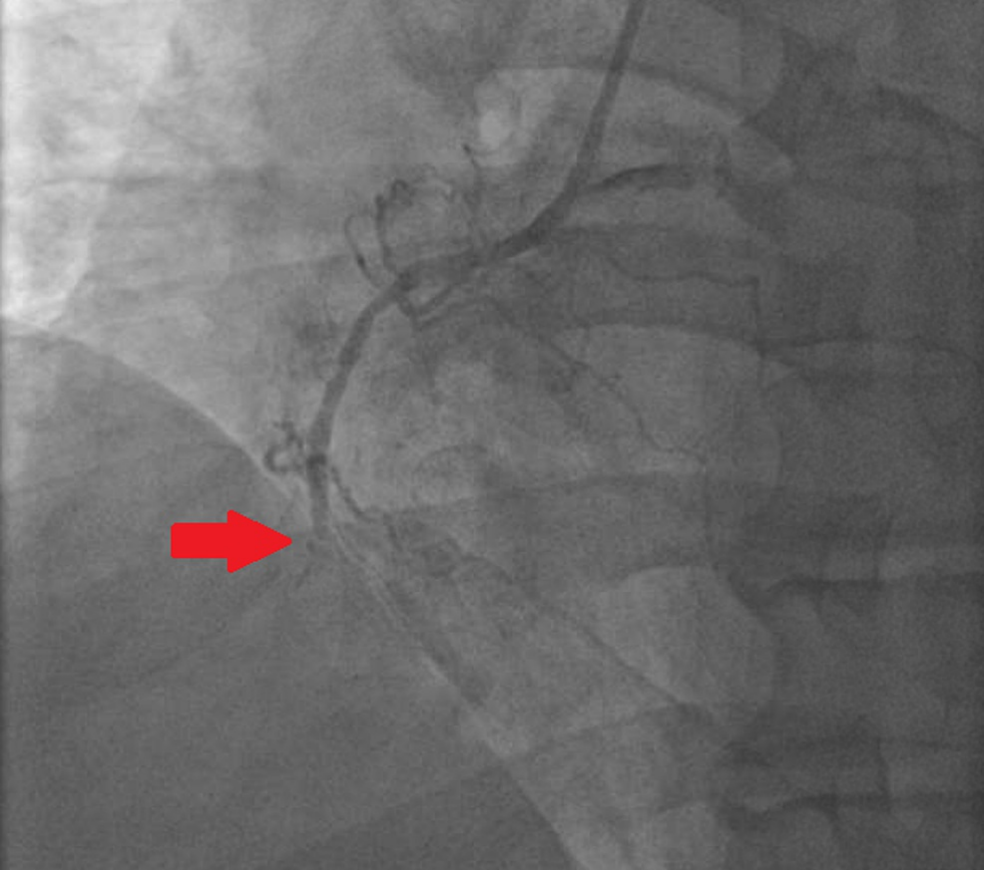
AP/LAO view showing 100% occlusion mid-RCA (arrow) AP: anterior-posterior; LAO: left anterior oblique; RCA: right coronary artery

**Figure 4:**
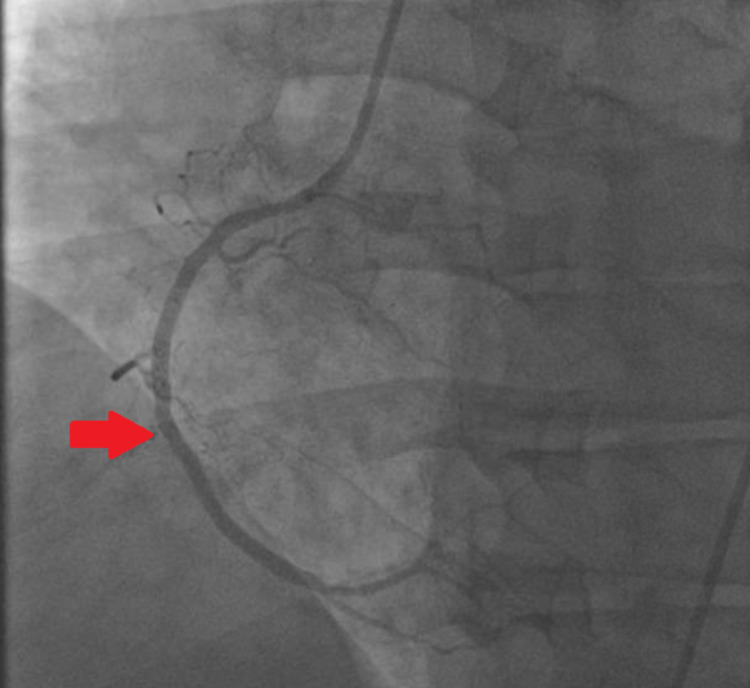
LAO/CRA view of the RCA after stent deployment LAO: left anterior oblique; CRA: cranial; RCA: right coronary artery

The patient was diagnosed with ST-elevation MI involving the inferior wall. Dopamine and norepinephrine were initiated. Emergent coronary angiography revealed 100% occlusion of the right coronary artery (RCA) with no collateral supply and 95% stenosis of the left anterior descending (LAD) artery (Figures 2, 3). Aspiration thrombectomy and balloon angioplasty and subsequent stenting of the RCA were performed (Figure 4).

Subsequent left ventriculography demonstrated evidence of a large ventricular septal defect (VSD) with extravasation of dye crossing from the left ventricle (LV) to the right ventricle (RV) near the apex (Video 1).

**Video 1:** Left ventriculography at LAO projection showing dye extravasation crossing from LV to RV LAO: left anterior oblique; LV: left ventricle; RV: right ventricle

A shunt run was performed to further confirm the presence of a VSD with the following results: right atrium (RA) saturation of 53.7%, RV: 90.4%, PA: 74.7%, and pulmonary capillary wedge pressure (PCWP): 95.8%. A notable step-up in the oxygen saturation between the RA and the RV was observed, consistent with a VSD.

Transthoracic echocardiogram with color Doppler was performed, which confirmed the presence of a defect in the septum. Color Doppler demonstrated a clear jet entering the ventricular septum from the LV, with the jet traversing the entire length of the septum through dissection and entering into the RV, consistent with complete IVSD (Video 2). 

**Video 2:** Transthoracic echocardiography showing apical four-chamber view demonstrating the complete dissection of IVS with color Doppler indicating flow from LV to RV through the dissected septum IVS: interventricular septum; LV: left ventricle; RV: right ventricle

The patient was maintained on cardiac pressors and was transferred to an outside hospital where he underwent successful bovine pericardial patch repair of the ventricular septum. The patient was ultimately discharged to a rehabilitation center in stable condition.

## Discussion

IVSD is a rare but potentially fatal complication of MI [1]. In one institutional review of 789,114 transthoracic echocardiograms, there were only 13 cases of IVSD [2]. Of these cases, 11 had involvement of SOV [2]. IVSD forms due to the dissection of the ventricular septum, which leads to an aneurism that is usually filled by one of the ventricles. The pathophysiology of the dissection is not very clear. However, a few different hypotheses have been proposed. One of the common hypotheses is that after the MI, the blood supply is compromised, especially in the septal perforator arteries [3]. Septal perforator arteries are vessels that arise from the right coronary and the LAD artery. The disruption in the blood supply causes opposing forces from the RCA and LAD on the ventricular septum, thereby resulting in the dissection [3]. The helical myocardial muscular band also plays a significant role in IVSD [2]. Other cases have been related to SOV aneurism [2,4].

In addition to the presentation of MI, typical symptoms of IVSD include hypotension and a harsh systolic murmur. IVSD can be diagnosed by transthoracic echocardiography, which generally shows an interventricular false cavity that is connected to the LV or RV [2,5]. MRI may also be used, and it normally shows the splitting of the septum [6]. Surgical repair is the treatment of choice. The ventricular wall may not be strong enough to be repaired after the dissection, necessitating a complete resection of the ventricular wall and repair of the VSD [7]. Aortic valve replacement is also commonly required in these cases [7].

## Conclusions

Although it is rare, physicians need to be aware of the possibility of the IVSD as a complication of inferior MI due to its high mortality rates. The presence of a systolic murmur is a huge clue that will help make the diagnosis. Evaluation with echocardiography will usually reveal the dissection and confirm the diagnosis. Surgical repair is the best way to resolve the problem.
